# Severe COVID-19: A Review of Recent Progress With a Look Toward the Future

**DOI:** 10.3389/fpubh.2020.00189

**Published:** 2020-05-13

**Authors:** Peng Xie, Wanyu Ma, Hongbo Tang, Daishun Liu

**Affiliations:** ^1^Department of Critical Care Medicine of the Third Affiliated Hospital (The First People's Hospital of Zunyi), Zunyi Medical University, Zunyi, China; ^2^Department of Emergency Intensive Care Unit of the Third Affiliated Hospital (The First People's Hospital of Zunyi), Zunyi Medical University, Zunyi, China; ^3^Department of Respiratory and Critical Medicine of the Third Affiliated Hospital (The First People's Hospital of Zunyi), Zunyi Medical University, Zunyi, China

**Keywords:** COVID-19, epidemiology, pathogenesis, diagnosis, treatment

## Abstract

The novel coronavirus disease 2019 (COVID-19) is an acute infectious disease caused by infection with severe acute respiratory syndrome coronavirus 2 (SARS-CoV-2). Currently, the World Health Organization has confirmed that COVID-19 is a global infectious disease pandemic. This is the third acute infectious disease caused by coronavirus infection in this century, after sudden acute respirator syndrome and Middle East respiratory syndrome. The damage mechanism of SARS-CoV-2 is still unclear. It is possible that protein S binds to angiotensin-converting enzyme 2 receptors and invades alveolar epithelial cells, causing direct toxic effects and an excessive immune response. This stimulates a systemic inflammatory response, thus forming a cytokine storm, which leads to lung tissue injury. In severe cases, the disease can lead to acute respiratory distress syndrome, septic shock, metabolic acidosis, coagulation dysfunction, and multiple organ dysfunction syndromes. Patients with severe COVID-19 have a relatively high mortality rate. Currently, there are no specific antiviral drugs for the treatment of COVID-19. Most patients need to be admitted to the intensive care unit for intensive monitoring and supportive organ function treatments. This article reviews the epidemiology, pathogenesis, clinical manifestations, diagnosis, and treatment methods of severe COVID-19 and puts forward some tentative ideas, aiming to provide some guidance for the diagnosis and treatment of severe COVID-19.

## Introduction

Since December 2019, several cases of pneumonia of unknown etiology with a history of exposure to the Huanan Seafood Wholesale Market in Wuhan, Hubei province, China, were discovered (1). On 11 February 2020, the International Committee on Taxonomy of Viruses named this virus severe acute respiratory syndrome coronavirus 2 (SARS-CoV-2) (2). On the same day, the World Health Organization (WHO) named the disease caused by SARS-CoV-2 as coronavirus disease-19 (COVID-19) (3). Currently, COVID-19 has become a public health emergency of international concern, and the WHO has upgraded its threat status to the “highest” level.

By 25 April 2020, 2,812,557 confirmed cases of COVID-19 were reported to the WHO, by 185 countries or regions, 197,217 of which resulted in death. The overall mortality rate was 7.01% (4). Although the major organ involved in COVID-19 is the lungs, the heart, kidneys, genitals, and liver are also damaged (5–7). A recent retrospective study found that the proportion of patients with severe COVID-19 who develop acute respiratory distress syndrome (ARDS), acute kidney injury, abnormal hepatic function, and cardiac injury are 67.3, 28.9, 28.9, and 23.1%, respectively, and the 28-day mortality rate is 61.5% (8). Due to the unique work nature of the intensive care unit (ICU), COVID-19 poses an immense challenge to medical staff in the ICU, as not only does it require an increase in manpower and materials but there is also a risk of infection (9). This article reviews the epidemiology, pathogenesis, clinical manifestations, diagnosis, and treatment methods of severe COVID-19, aiming to provide some guidance for the diagnosis and treatment of severe COVID-19.

## Epidemiology

### Pathogen

SARS-CoV-2 is an animal virus that belongs to the β-coronavirus genus (10). Current studies showed that bats, snakes, and pangolins may be the hosts for SARS-CoV-2 (11–13). However, analysis results of whole genome sequencing showed bats as the host for this virus as the homology between SARS-CoV-2 and bat coronaviruses is 96% (11). Regrettably, the intermediate host for SARS-CoV-2 is still unknown.

### Source of Infection and Transmission Routes

Presently, the main source of infection is patients with COVID-19, and asymptomatic patients can become sources of infection (14, 15). Respiratory droplets and close contact are the main transmission routes, and particular attention should be paid to family and asymptomatic transmission (14). Currently, SARS-CoV-2 has been detected in the air in the ICU, and long-term exposure in the relatively sealed ICU environment may lead to aerosol transmission. Additionally, SARS-CoV-2 has also been detected in the gastrointestinal tract, urine, saliva, and tears of patients with COVID-19 (14, 16, 17). Moreover, China has reported infants with a confirmed diagnosis of COVID-19 3 days after birth, suggesting the possibility of vertical transmission. Therefore, ICU medical staff should conduct preventive measures to reduce nosocomial infection as much as possible.

### Pathogenesis

Currently, pathogenesis of COVID-19 is still unclear, and the following factors may be involved: (1) SARS-CoV-2 binds to the angiotensin-converting enzyme-2 (ACE2) receptor through the coronavirus spike (S) protein to invade alveolar epithelial cells to promote direct toxicity and excessive immune responses. The induced systemic inflammation causes a cytokine storm, resulting in lung injury, and patients with severe disease develop respiratory failure and die (18–22). (2) Pathological results found that the lungs of patients with COVID-19 show diffuse alveolar damage and hyaline membrane formation in the lungs, and the overall pathological presentation of the lungs is similar to that in SARS and Middle East respiratory syndrome (MERS) (23). (3) ACE2 is also expressed in the kidneys, heart, lung, and intestines, and SARS-CoV-2 can invade cells in the aforementioned tissues to proliferate and destroy these organs, leading to multiple organ dysfunction syndrome (MODS) (24, 25). (4) Levels of IL-2, IL-6, IL-7, IL-10, granulocyte colony-stimulating factor, interferon gamma-induced protein 10, monocyte chemoattractant protein-1, macrophage inflammatory protein 1α, and tumor necrosis factor α are significantly elevated in patients with severe COVID-19, which may be associated with poor outcomes (26, 27). (5) Excessive activation of lymphocytes in patients with COVID-19 and an increase in pro-inflammatory CCR4+CCR6+Th17 cells promotes immune-mediated damage, which causes a mild disease to increase in severity, and single organ involvement to progress to MODS. In particular, elderly individuals with reduced immunity and patients with comorbidities are more susceptible to infection (20).

### Clinical Presentation and Auxiliary Tests

Based on previous studies (5, 8, 14) and our ICU observations, patients with severe COVID-19 mostly develop dyspnea and/or hypoxemia 1 week after disease onset, and more severe cases can rapidly progress to ARDS, septic shock, refractory metabolic acidosis, coagulation disorder, and MODS. Additionally, patients with COVID-19 and comorbid encephalitis should not be overlooked, as cerebral congestion and edema and neuropathy may develop in these patients, and attention should be paid to neurological symptoms in clinical practice. Initial neurological symptoms have been reported in some patients affected by COVID-19, such as dizziness, headache, anosmia, myalgia, impaired consciousness, and acute cerebrovascular diseases (28–31). Future studies should elucidate the incidence of these neurological complications and their therapeutic options.

Auxiliary markers that predict severe COVID-19 are as follows: (1) progressive decline in peripheral blood lymphocyte count; (2) progressive elevation in peripheral blood inflammatory factors such as IL-6 and the C-reactive protein; (3) progressive elevation in lactic acid level; (4) and imaging results showing bilateral or multilobar infiltration, pleural effusion, or short-term increase in lesions (32, 33). Interestingly, some researchers found that the neutrophil-to-lymphocyte ratio (NLR) is an influencing factor that can be used for early identification of the prognosis of patients with severe COVID-19. Patients aged ≥50 years and with NLR ≥3.13 tend to develop severe COVID-19 and should be admitted to the ICU immediately (34). Lastly, it should be pointed out that chest CT plays an extremely crucial role in COVID-19 diagnosis and the disease severity assessment. Chest CT has high diagnostic value in patients who have negative Reverse Transcription-Polymerase Chain Reaction (RT-PCR) results but whose clinical symptoms, auxiliary test results, and epidemiological history make them highly suspected patients (35).

### Diagnosis of Severe COVID-19

Diagnosis of COVID-19 and compliance with any one of the following can be diagnosed as severe COVID-19 (8, 14, 34): (1) respiratory distress, respiratory rate ≥30 breaths/min; (2) pulse oximetry oxygen saturation at rest ≤ 93%; (3) oxygenation index (PaO_2_/FiO_2_) ≤ 300 mmHg (1 mmHg = 0.133 kPa); (4) lung imaging tests showing significant progression (>50%) in lesions in 24–48 h; (5) age ≥50 years and NLR ≥3.13; (6) respiratory failure and need for mechanical ventilation (non-invasive or invasive ventilator); (7) shock; and (8) comorbid failure in other organs and need for ICU monitoring and treatment.

### Treatment

Treatment of severe COVID-19 includes aggressive treatment of complications, prophylaxis for secondary infection, and organ function support based on treatment of underlying disease.

#### Antiviral Drugs

Currently, there are no specific antiviral drugs for COVID-19. Moreover, remdesivir, lopinavir, and ritonavir may be effective against COVID-19 (26, 36), but their efficacy and safety still require a large sample size for clinical validation. Furthermore, interferon-α nebulization, ribavirin, chloroquine, and umifenovir are also used in anti-SARS-CoV-2 treatment. Regardless of the antiviral drug used, it should be immediately discontinued when the patient develops coughing, nausea, vomiting, diarrhea, rashes, liver impairment, and other adverse reactions or intolerable toxic side effects.

#### Respiratory Support

The invasive mechanical ventilation rates of severe COVID-19, SARS and MERS are 42, 76, and 85%, respectively (9). Studies showed that most patients with COVID-19 die of respiratory failure (5, 37). Therefore, respiratory support is the mainstay treatment for severe COVID-19. When respiratory distress and/or hypoxemia cannot be alleviated after standard oxygen therapy in patients with severe COVID-19, it is recommended that transnasal high-flow oxygen or non-invasive ventilation be used. If the patient's condition does not improve or even worsens within a short time, endotracheal intubation and invasive mechanical ventilation should be immediately performed. Table 1 shows the ventilation options: For conservative oxygen therapy, the target SpO_2_ value is 88–92%, low tidal volume ventilation is 4–8 mL/kg, and respiratory rate is 18–25 breaths/min. Positive end expiratory pressure (PEEP) ventilation should be adjusted according to ARDS severity or titration or patient's response to PEEP (whether oxygenation or compliance improves). When the oxygenation index is <100 mmHg, ventilation should be performed in a prone position. Airway management is especially critical in severe COVID-19 as there is low mucus production in the airway in patients and viscosity is high. In clinical practice, we also observed that it is extremely difficult for nurses to perform sputum suction. We recommend that a fiberoptic bronchoscope be used for sputum suction or bronchoalveolar lavage when necessary.

**Table 1:** Respiratory supportive treatment for COVID-19.

**Support method**	**Strategy**
Does not need oxygen	SpO_2_ of >93% and absence of apparent respiratory distress symptoms
Oxygen therapy	R of ≥30 breaths/min and/or SpO_2_ of ≤ 93% on breathing
High-flow oxygen therapy	Respiratory failure and mild-moderate ARDS (150 mmHg < PaO_2_/FiO_2_ ≤ 300 mmHg), HFNO therapy is used as first-line treatment, followed by NIV
Non-invasive ventilation	NIV is not recommended for patients with failed HFNO treatment
	Benefits patients with mild-moderate ARDS, which is mainly presented as providing PEEP, and reduces the respiratory load and intubation rate
Invasive ventilation	Unstable hemodynamics, persistent non-improvement of PaO_2_/FiO_2_, R of >40 breaths/min, significant acidosis, and large volumes of airway secretions
	ROX index of <3.85 after 12 h of HFNO support; PaO_2_/FiO_2_ of <150 mmHg after 2 h of HFNO or NIV support
	Mask oxygen therapy (flow rate: 10–15 L/min), SpO_2_ of ≤ 90%, R of ≥30 breaths/min, and respiratory support should be provided as soon as possible
	Invasive ventilation is recommended for patients with moderate-severe ARDS (PaO_2_/FiO_2_ ≤ 150 mmHg) or patients with failed HFNO and NIV treatment
PVS	Tidal volume: 4–8 mL/kg, respiratory rate: 18–25 breaths/min, adjusting it according to pause pressure and PaCO_2_
PEEP	PEEP is adjusted according to the severity of ARDS (mild: 5–7 cmH_2_O, moderate: 8–12 cmH_2_O, and severe: >12 cmH_2_O), or titration can be performed in accordance with the patient's response to PEEP ventilation.
	The use of PEEP titration is recommended to set the appropriate PEEP level. A recommended table can be used for PEEP titration. If SPO_2_ is >93%, PEEP should be decreased.
Lung recruitment	When FiO_2_ is >0.06, recruitment evaluation is recommended, and limited-pressure lung recruitment should be carried out in recruitable patients
Prone position	The prone position when PaO_2_/FiO_2_ is <100 mmHg
	The prone position for >12 h as soon as possible is recommended for patients with moderate-severe ARDS (PaO_2_/FiO_2_ ≤ 150 mmHg)

*SpO_2_, blood oxygen saturation; R, respiratory rate; PaO_2_/FiO_2_, oxygenation index; HFNO, high-flow nasal oxygen; NIV, non-invasive ventilation; ARDS, acute respiratory distress syndrome; PEEP, positive end-expiratory pressure; ROX: [SpO_2_/(FiO_2_ × RR), PaCO_2_], partial pressure of carbon dioxide; FiO_2_, fraction of inspired oxygen; PVS, Protective ventilation strategy*.

#### Circulatory Support and Myocardial Protection

When shock occurs in patients with severe COVID-19, aggressive hemodynamic and metabolic marker monitoring must be conducted, and hemodynamic disorder should be corrected as soon as possible to improve oxygen supply to tissues, protect organ function, and to prevent the development of MODS. Conservative fluid treatment strategies are recommended for fluid resuscitation in patients with severe COVID-19. This will not only improve lung function and shorten mechanical ventilation duration and length of ICU stay in patients with acute lung injury, but will also prevent extrapulmonary organ failure (38). Simultaneously, if shock is not corrected after fluid resuscitation, vasoactive drugs should be used. Norepinephrine or dopamine can be selected based on the patient's condition. If reduced systolic function is present, dobutamine can be used depending on the situation (39). Creatine sodium phosphate, vitamin C, coenzyme Q, and polarized solution can be used depending on the situation when comorbid myocardial injury is present in severe COVID-19. Troponin I/T and B-type natriuretic peptide should be checked daily as a warning signal for acute fulminant myocarditis.

#### Extracorporeal Membrane Oxygenation Treatment

Although it is still controversial whether Extracorporeal membrane oxygenation (ECMO) can improve the prognosis of patients with severe ARDS (40, 41), recent studies on MERS showed that ECMO can be used as a salvage treatment to reduce the mortality rate of refractory hypoxemia (42). Based on similar principles, ECMO may also be an effective treatment for severe COVID-19 (43, 44). When severe ARDS occurs in severe COVID-19 and outcomes of aggressive respiratory support, lung recruitment, and ventilation in the prone position are poor (oxygenation index <100 mmHg or PaCO_2_ >50 mmHg and pH <7.25 or pause pressure > 35 cmH_2_O), ECMO should be considered as soon as possible. However, ECMO may stimulate the release of cytokines and exacerbate inflammatory responses in patients with COVID-19. Therefore, continuous renal replacement therapy (CRRT) should be considered when using ECMO treatment (45).

#### CRRT and Artificial Liver Support Therapy

CRRT treatment should be performed as soon as possible in patients with severe COVID-19 with excessive inflammatory responses. The treatment options include plasma replacement, blood adsorption, and perfusion. If liver failure is present in patients with severe COVID-19, an artificial liver blood purification system can be used for treatment. From the treatment experiences in China, CRRT and artificial liver support therapy can shorten the length of ICU stay and reduce serum levels of cytokines, such as IL-2, IL-4, IL-6, and TNF-α.

#### Steroid and Traditional Chinese Medicine Treatment

Previous studies showed that glucocorticoids can reduce the mortality rate of patients with SARS (46), but some researchers found that glucocorticoids will not only increase the mortality rate of patients with SARS but also delay viral clearance in MERS and SARS (47–49). Therefore, there is an ongoing debate on the use of glucocorticoid treatment in severe viral pneumonia. We do not recommend glucocorticoid use in the treatment of mild COVID-19. However, low doses of glucocorticoids can be used in the short term in patients with progressive worsening of oxygenation markers, rapid progressive imaging, and excessive inflammatory responses. In view of the pathological presentation of pulmonary edema and hyaline membrane formation in patients with COVID-19 on autopsy (23), glucocorticoids should be considered in severe COVID-19 to prevent ARDS progression. Many studies have shown that traditional Chinese medicine plays an important role in the treatment of COVID-19, which brings hope for the prevention and control of COVID-19 (50–52). Refer to China's protocols for traditional Chinese medicine treatment, such as the use of Shuanghuanglian oral liquid, Xuebijing, Xiyanping, Reduning, and Xingnaojing injections.

#### Maintenance of Gastrointestinal Function and Nutritional Supportive Treatment

A study has shown that gastrointestinal epithelial cells contain large amounts of ACE2, and SARS-CoV-2 can invade the intestinal tract through ACE2 receptors in these cells, resulting in gastrointestinal dysfunction and changes in the gut microbiota (53). High inflammatory responses disrupt the intestinal barrier and increases permeability, causing bacterial translocation into the circulation and secondary systemic infection (54). Simultaneously, the influx of large amounts of lipopolysaccharides causes the release of TNFα, IL-1β, and IL-6, further exacerbating systemic inflammation (55). Patients with respiratory tract infection often develop intestinal dysfunction, and gut microbiota dysregulation exacerbates lung injury. Gut and respiratory tract flora interfere with each other (53), and a study showed that regulating gut microbiota can reduce the development of enteritis and ventilator-associated pneumonia (56). Therefore, it is particularly important to administer probiotics to patients with COVID-19 to maintain the equilibrium of the gut microflora and ameliorate gastrointestinal symptoms to prevent secondary bacterial infection. We recommend that rational nutritional support be provided to patients with severe COVID-19, including sufficient energy, amino acid, and trace elements to improve immunity and to regulate gut microbiota dysregulation.

#### Treatment With Plasma From Recovered Patients

Evidence has shown that plasma from recovered patients can be an effective treatment for MERS and SARS and can significantly help reduce the mortality rate (57–59). After SARS-CoV-2 infection, the body generates immune responses to produce corresponding specific antibodies. Before treatment with non-specific antiviral drugs, plasma from recovered patients can be used to treat patients with severe COVID-19 (60, 61). Currently, we have conducted relevant clinical trials and are awaiting subsequent observations for efficacy evaluation. However, plasma from recovered patients is currently available for empirical use, and it is necessary to understand the indications, closely monitor the transfusion process, and to perform dynamic evaluations (62).

### Prevention of ICU-Related Complications

Owing to the uniqueness of the ICU environment and patients' fear of the disease, detailed strategies for patient management should be formulated with particular attention to early sleep management, conducting humanistic care and rehabilitation training, and prevention of the occurrence of complications such as delirium, ICU-acquired weakness, and post-ICU syndrome.

### Criteria for ICU Discharge

**Figure 1:**
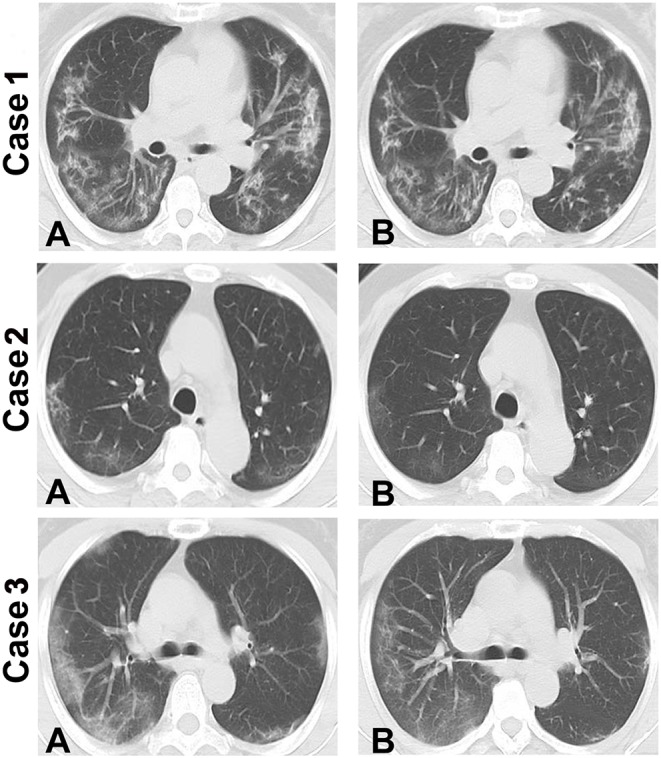
Chest CT showing changes in 3 patients with severe COVID-19. Compared to the first transferred to ICU, chest CT showing significant absorption of exudative lesions in patient of the day before they were discharged from the ICU. **(A)** Chest CT images of the patients when they were first transferred to ICU. **(B)** Chest CT images of the patients on the day before they were discharged from the ICU showing absorption of the exudative lesions. Intensive care unit: ICU.

The criteria for ICU discharge includes absence of fever for 3 days or more, significant improvement in respiratory symptoms, chest CT showing significant absorption of exudative lesions (Figure 1), negative results from 2 consecutive tests for respiratory pathogen nucleic acid (at least 1 day between tests), and absence of a life-threatening major organ impairment. After meeting the criteria, patients can be transferred to the corresponding department for treatment.

### Self-Protection of Medical Staff

The ICU is an important site for concentrated treatment of patients with COVID-19 and is a relatively closed space. Medical staff not only have to manage the possibility of many transmission routes for the virus, such as body fluids, secretions, and excretions from patients but also face the possibility of aerosol infection, particularly when performing endotracheal intubation, tracheotomy, fiberoptic bronchoscope sputum suction and bronchoalveolar lavage, and nebulization. Therefore, tertiary protective measures must be followed strictly. In addition, the number of ICU physicians and nurses during a shift should be increased, and the shift duration should be strictly controlled to ensure that medical staff have sufficient rest. The dietary structure should be rationally allocated to ensure sufficient nutrition and to maintain a healthy emotional state. Psychological counseling should be provided when necessary.

## Summary

Reducing the mortality rate is the primary goal for patients with severe COVID-19. In the absence of specific antiviral drugs, aggressive maintenance of organ function is a mainstay treatment. In the future, treatment protocols to improve the cure rates should be further optimized, and a vaccine should be actively developed for COVID-19. Owing to the uniqueness of the ICU environment, medical staff should perform strict self-protection.

## Author Contributions

PX, WM, and HT performed the literature search and wrote the first draft of the manuscript, which was critically reviewed by DL.

## Conflict of Interest

The authors declare that the research was conducted in the absence of any commercial or financial relationships that could be construed as a potential conflict of interest.
